# Supplementary material for: Influence of Electrolyte Choice on Zinc Electrodeposition
Source: Materials (Basel). 2024 Feb 10;17(4):851. doi: 10.3390/ma17040851 (PMC10890548; doi:10.3390/ma17040851)
Supplement: Supplementary file 1 [file materials-17-00851-s001.zip › materials-2753249-supplementary.pdf]

materials

Supplementary Materials

Influence of Electrolyte Choice on Zinc Electrodeposition

Kranthi Kumar Maniam [1,*], Corentin Penot [1] and Shiladitya Paul [1,2,*]

[1] Materials Innovation Centre, School of Engineering, University of Leicester, Leicester LE1 7RH, UK; cp473@leicester.ac.uk
[2] Materials Performance and Integrity Technology Group, TWI, Cambridge CB21 6AL, UK
* Correspondence: km508@leicester.ac.uk (K.K.M.); shiladitya.paul@twi.co.uk (S.P.)

1. Materials and Methods

Three distinct electrolyte solutions were prepared in-house for the electrodeposition of zinc, each with a unique composition and purpose: i) halide-free alkaline aqueous zinc (conventional), ii) choline-chloride-based ionic liquid (IL), and iii) halide-free acetate-based organic solution.

1.1. Halide-Free Acetate-Based Organic Solution:

The halide-free acetate solution was prepared by combining anhydrous ethylene glycol (EG, $C_2H_6O_2$, Alfa Aesar 99.8%) and potassium acetate (CH_3COONa, Fisher Scientific). The procedure followed was based on the method reported by Panzeri et al. [1], with modifications involving the use of potassium acetate as the conducting salt. Initially, EG was heated to 60 °C, and then a 0.5 M solution of potassium acetate was slowly added while maintaining the solution temperature at 60 °C. Next, zinc acetate ($Zn(CH_3COO)_2{\cdot}2H_2O$, Alfa Aesar) with a concentration of 0.75 M was introduced into the solution, and thorough mixing was performed to achieve a homogeneous mixture.

1.2. Choline-Chloride-Based Ionic Liquid (IL)

The choline-chloride-based IL was prepared by combining choline chloride (ChCl) and ethylene glycol (EG) in a 1:2 molar ratio. The preparation process followed the procedure reported by Abbott et al. Abbott et al.[2,3]. A 0.75 M solution of zinc chloride ($ZnCl_2$, A) was subsequently added to the mixture, and thorough mixing was carried out to ensure homogeneity.

1.3. Conventional Halide-Free Alkaline Aqueous Solution:

The conventional halide-free alkaline aqueous solution was prepared by mixing 12 g/L of zinc oxide (ZnO) and 120 g/L of sodium hydroxide (NaOH). This mixing process was carried out at 50 °C to ensure the formation of a homogeneous mixture.

Photographs of the prepared solutions are shown in figure S1 (supplementary information)

Citation: Maniam, K.K.; Penot, C.; Paul, S. Influence of Electrolyte Choice on Zinc Electrodeposition. *Materials* **2024**, *17*, x. https://doi.org/10.3390/xxxxx

Academic Editor: Masato Sone

Received: 18 November 2023
Revised: 3 February 2024
Accepted: 6 February 2024
Published: date

Copyright: © 2024 by the authors. Submitted for possible open access publication under the terms and conditions of the Creative Commons Attribution (CC BY) license (https://creativecommons.org/licenses/by/4.0/).

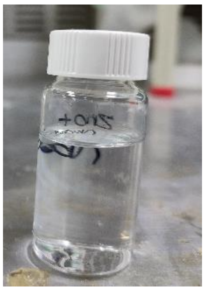

(a)

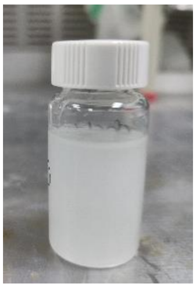

(b)

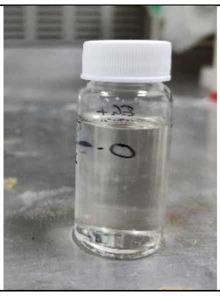

(c)

Figure S1. Photographs of the prepared electrolyte solutions prior to deposition: (**a**) halide free alkaline (conventional); (**b**) choline chloride based DES and (c) acetate based organic solution.

3. Results

In this study, a comparative analysis of three different electrolytes was conducted to gain valuable insights into their performance during zinc electrodeposition. The results were visually represented in Figure S2 of the supplementary information, and representative optical micrographs were displayed in Figure S3. One noteworthy aspect of this research is its practical relevance to industrial applications. Traditional water-based electrolytes have long been the standard, but organic solutions have emerged as a promising alternative. To evaluate their suitability for industrial use and achieve a practical assessment, hull cell tests were employed. Considering the nature of the solutions presented in this study, an unconventional glass tenori cell, with a volume of 33 ml, was selected for this purpose. This cell allowed for a wide range of current density distribution, spanning from 1 mA.cm^{-2} to 20 mA.cm^{-2}, with a total cathodic current of 0.1 A. The current distribution in the cell was described by an empirical law (Equation (1)):

$$C:D. = I[C1 - C2Log(L)] \quad (1)$$

Here, C.D. represents the cathodic current density in A.dm^{-2}, while C1 and C2 are constants dependent on the electrolyte properties. L (cm) denotes the distance from the

high-current side of the cathode. For this specific cell set up, the constants were determined as C1 = 13.88 and C2 = 20.55 by the manufacturer

Interestingly, the results showed that while there was some slight burning observed at high current density areas near the anode, a bright deposit was consistently observed across the entire tenori hull cell for zinc deposition using the three different electrolytes. Notably, when the zinc was deposited at an applied current density of 10 $mA.cm^{-2}$ for both 15 minutes and 30 minutes, the resulting deposits exhibited a bright and visually similar appearance, indicating promising results for all three electrolytes under consideration.

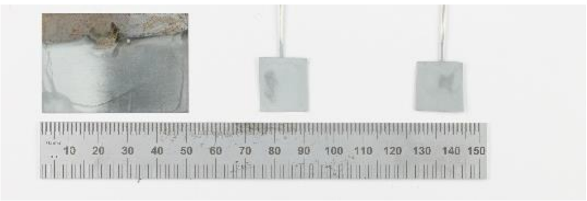

(a)

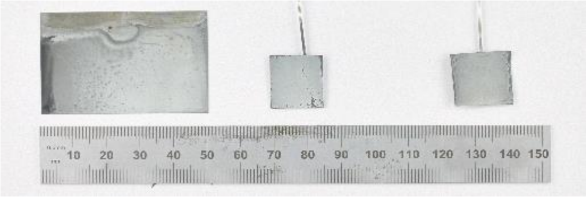

(b)

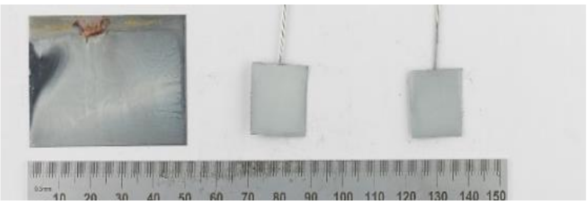

(c)

Figure S2. Photographs of the deposited Zn coatings using (**a**) halide free alkaline (conventional); (**b**) choline chloride based DES and (c) acetate based organic solution. Photographs (left) – tenori hull cell panel after zinc deposition; (middle) –zinc deposited for 15 minutes and (right) – zinc deposited for 30 minutes

Uniform, smooth and homogeneous coatings were observed in all the cases.

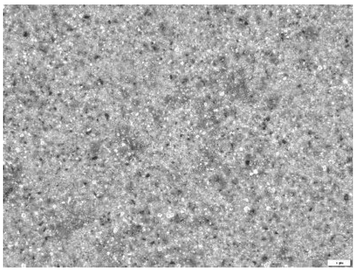

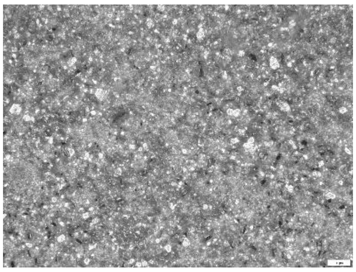

(a)

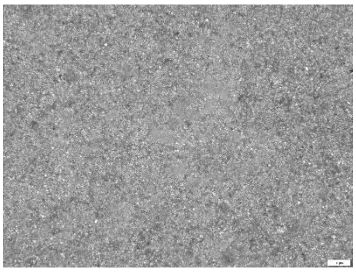

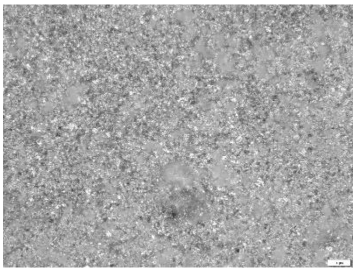

(b)

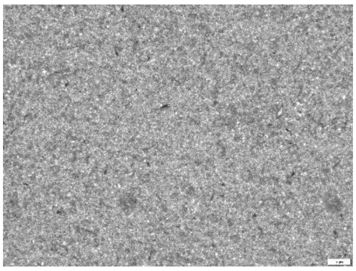

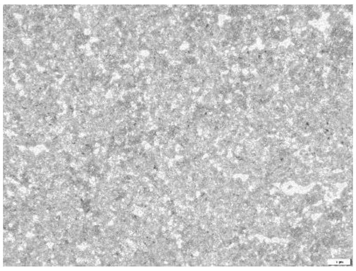

(c)

Figure S3. Optical micrographs of the deposited Zn coatings using (a) halide free alkaline (conventional); (b) choline chloride based DES and (c) acetate based organic solution. (left) –zinc deposited for 15 minutes and (right) – zinc deposited for 30 minutes

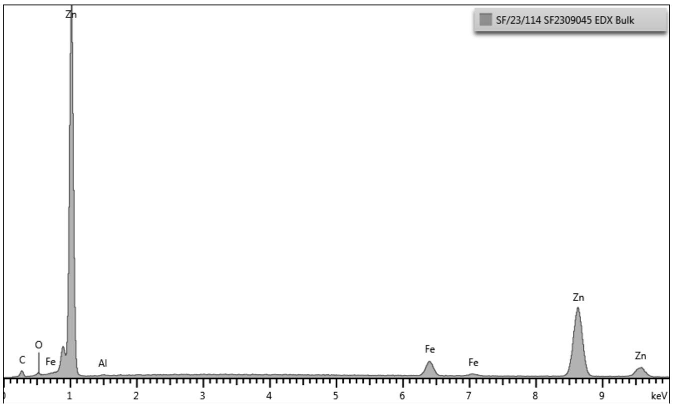

(a)

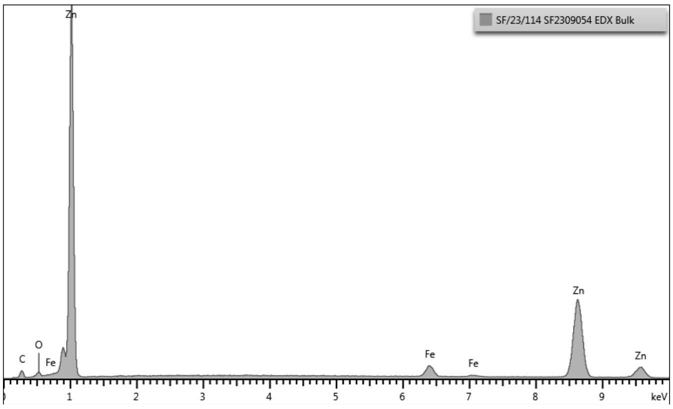

(b)

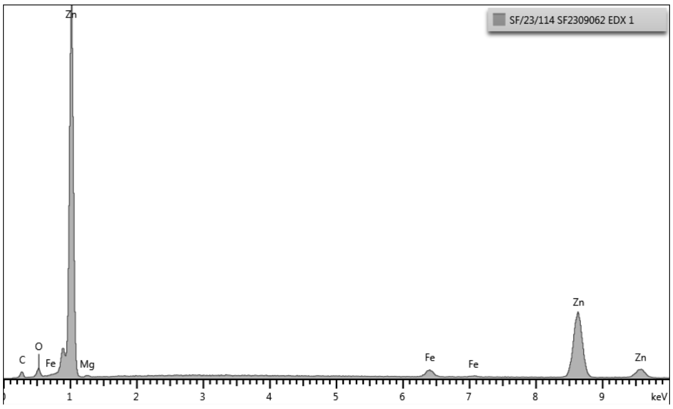
SF/23/114 SF2309062 EDX 1
Zn
O
C
Fe
Mg
Fe
Fe
Zn
Zn
1 2 3 4 5 6 7 8 9 keV

(c)

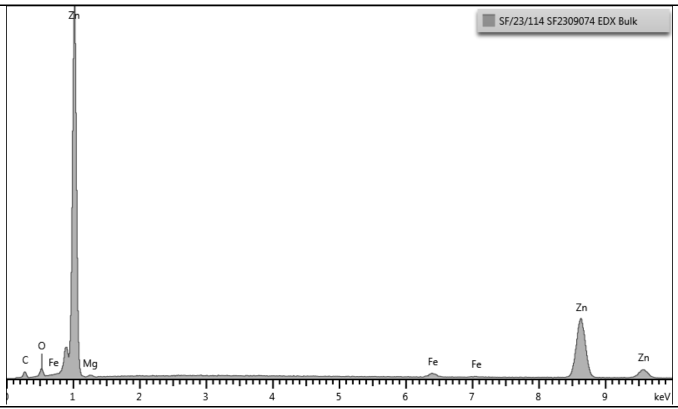
SF/23/114 SF2309074 EDX Bulk
Zn
O
C
Fe
Mg
Fe
Fe
Zn
Zn
1 2 3 4 5 6 7 8 9 keV

(d)

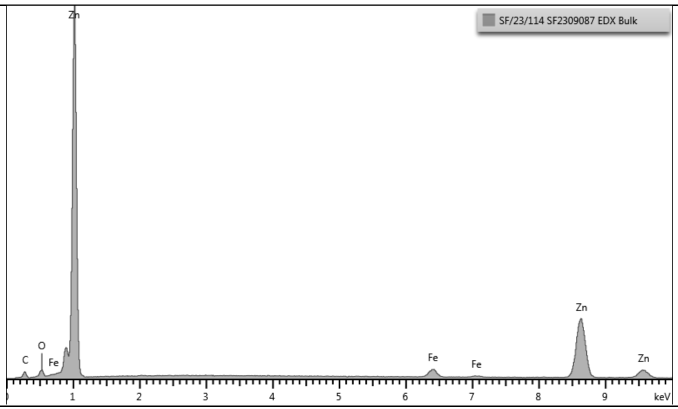
SF/23/114 SF2309087 EDX Bulk
Zn
O
C
Fe
Fe
Fe
Zn
Zn
1 2 3 4 5 6 7 8 9 keV

(e)

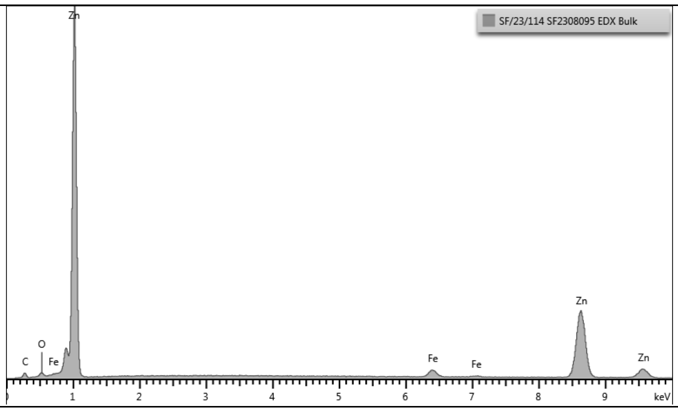
SF/23/114 SF2308095 EDX Bulk
Zn
O
C
Fe
Fe
Fe
Zn
Zn
1 2 3 4 5 6 7 8 9 keV

(f)

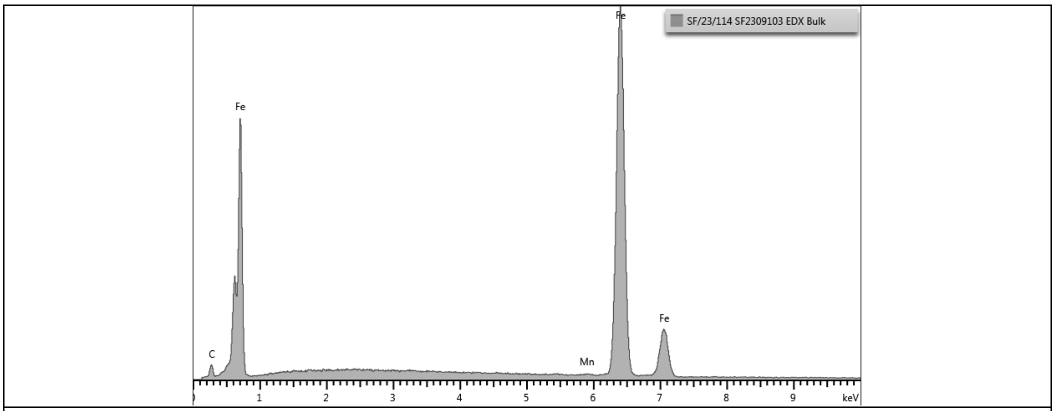

(g)

Figure S4. Energy dispersive spectrum (EDS) of the zinc coatings obtained using three different electrolytes: halide free alkaline (conventional); choline chloride-based DES and acetate based organic solution for a deposition time of 15 minutes (a, b, c) and 30 minutes (d, e, f). (g) represents the EDS spectrum of the bare mild steel substrate.

Supplementary Materials: The following supporting information can be downloaded at: www.mdpi.com/xxx/s1, Figure S1: Photographs of the prepared electrolyte solutions prior to deposition: (**a**) halide free alkaline (conventional); (**b**) choline chloride based DES and (c) acetate based organic solution; Figure S2: Photographs of the deposited Zn coatings using (**a**) halide free alkaline (conventional); (**b**) choline chloride based DES and (c) acetate based organic solution. Photographs (left) – tenori hull cell panel after zinc deposition; (middle) –zinc deposited for 15 minutes and (right) – zinc deposited for 30 minutes; Figure S3: Optical micrographs of the deposited Zn coatings using (a) halide free alkaline (conventional); (b) choline chloride based DES and (c) acetate based organic solution. (left) –zinc deposited for 15 minutes and (right) – zinc deposited for 30 minutes; Figure S4: Energy dispersive spectrum (EDS) of the zinc coatings obtained using three different electrolytes: halide free alkaline (conventional); choline chloride-based DES and acetate based organic solution for a deposition time of 15 minutes (a, b, c) and 30 minutes (d, e, f). (g) represents the EDS spectrum of the bare mild steel substrate.

Author Contributions: Conceptualization, K.K.M and S.P.; methodology, K.K.M.; formal analysis, K.K.M and C.P; investigation, K.K.M.; resources, K.K.M. and C.P.; writing—original draft preparation, K.K.M. and C.P.; writing—review and editing, K.K.M. and S.P.; supervision, S.P. All authors have read and agreed to the published version of the manuscript.

Funding: This research has received funding from the European Union's Horizon 2020 research and innovation program under the Marie Sklodowska-Curie grant agreement No.885793.

Institutional Review Board Statement: Not applicable

Informed Consent Statement: Not applicable

Data Availability Statement: Not applicable

Conflicts of Interest: The authors declare no conflict of interest.

References

References must be numbered in order of appearance in the text (including citations in tables and legends) and listed individually at the end of the manuscript. We recommend preparing the references with a bibliography software package, such as EndNote, ReferenceManager or Zotero to avoid typing mistakes and duplicated references. Include the digital object identifier (DOI) for all references where available.

Citations and references in the Supplementary Materials are permitted provided that they also appear in the reference list here.

In the text, reference numbers should be placed in square brackets [ ] and placed before the punctuation; for example [1], [1–3] or [1,3]. For embedded citations in the text with pagination, use both parentheses and brackets to indicate the reference number and page numbers; for example [5] (p. 10), or [6] (pp. 101–105).

1. Panzeri, G.; Muller, D.; Accogli, A.; Gibertini, E.; Mauri, E.; Rossi, F.; Nobili, L.; Magagnin, L. Zinc electrodeposition from a chloride-free non-aqueous solution based on ethylene glycol and acetate salts. *Electrochim. Acta* **2019**, *296*, 465–472, doi:10.1016/j.electacta.2018.11.060.
2. Abbott, A.P.; Barron, J.C.; Frisch, G.; Ryder, K.S.; Silva, A.F. The effect of additives on zinc electrodeposition from deep eutectic solvents. *Electrochim. Acta* **2011**, *56*, 5272–5279, doi:10.1016/j.electacta.2011.02.095.
3. Abbott, A.P.; Boothby, D.; Capper, G.; Davies, D.L.; Rasheed, R.K. Deep Eutectic Solvents Formed between Choline Chloride and Carboxylic Acids: Versatile Alternatives to Ionic Liquids. *J. Am. Chem. Soc.* **2004**, *126*, 9142–9147, doi:10.1021/ja048266j.

Disclaimer/Publisher's Note: The statements, opinions and data contained in all publications are solely those of the individual author(s) and contributor(s) and not of MDPI and/or the editor(s). MDPI and/or the editor(s) disclaim responsibility for any injury to people or property resulting from any ideas, methods, instructions or products referred to in the content.
